# National consensus recommendations on patient-centered care for ductal carcinoma in situ

**DOI:** 10.1007/s10549-019-05132-z

**Published:** 2019-01-09

**Authors:** Anna R. Gagliardi, Frances C. Wright, Nicole J. Look Hong, Gary Groot, Lucy Helyer, Pamela Meiers, May Lynn Quan, Robin Urquhart, Rebecca Warburton

**Affiliations:** 1 Toronto General Hospital Research Institute, University Health Network, 200 Elizabeth Street, Toronto, M5G2C4 Canada; 2 Odette Cancer Research Program, Sunnybrook Health Sciences Centre, Toronto, Canada; 3 General Surgery & Community Health and Epidemiology, University of Saskatchewan, Saskatoon, Canada; 4 Department of Surgery, Dalhousie University, Nova Scotia, Canada; 5 Irene and Leslie Dubé Centre of Care Breast Health Centre, Saskatoon City Hospital, Saskatoon, Canada; 6 Calgary Breast Health Program Foothills Medical Centre, University of Calgary, Calgary, Canada; 7 Department of Surgery, University of British Columbia, Vancouver, Canada

**Keywords:** Ductal carcinoma in situ, Consensus, Recommendations, Patient-centered care

## Abstract

**Purpose:**

The purpose of this research was to generate recommendations on strategies to achieve patient-centered care (PCC) for ductal carcinoma in situ (DCIS).

**Methods:**

Thirty clinicians (surgeons, medical/radiation oncologists, radiologists, nurses, navigators) who manage DCIS and 32 DCIS survivors aged 18 or older were nominated. Forty-six recommendations to support PCC for DCIS were derived from primary research, and rated in a two-round Delphi process from March to June 2018.

**Results:**

A total of 29 clinicians and 27 women completed Round One, and 28 clinicians and 22 women completed Round Two. The 29 recommendations retained by both women and clinicians reflected the PCC domains of fostering patient–physician relationship (5), exchanging information (5), responding to emotions (1), managing uncertainty (4), making decisions (9), and enabling patient self-management (5). An additional 13 recommendations were retained by women only: fostering patient–physician relationship (1), exchanging information (3), responding to emotions (2), making decisions (3), and enabling patient self-management (4). Some recommendations refer to processes (i.e., ask questions about lifestyle or views about risks/outcomes to understand patient preferences); others to tools (i.e., communication aid). Panelists recommended a separate consensus process to refine the language that clinicians use when describing DCIS.

**Conclusions:**

This is the first study to generate guidance on how to achieve PCC for DCIS. Organizations that deliver or oversee health care can use these recommendations on PCC for DCIS to plan, evaluate, or improve services. Ongoing research is needed to develop communication tools, and establish labels and language for DCIS that optimize communication.

**Electronic supplementary material:**

The online version of this article (10.1007/s10549-019-05132-z) contains supplementary material, which is available to authorized users.

## Introduction

Approximately 15–25% of mammographically detected lesions are ductal carcinoma in situ (DCIS) [1]. The incidence of DCIS is increasing globally concomitant with rising mammography rates [1, 2]. DCIS is a complex premalignant disease that includes a spectrum of abnormal cell types confined to the breast ducts with variable natural history, and risk of progression and recurrence [1]. Approximately 20% of cases will progress to invasive disease so most women with DCIS will never develop breast cancer and have a favorable prognosis, although DCIS may be more aggressive in women less than 50 years of age and African American women [2]. The 20-year breast cancer-specific mortality is 3.3% [2]. However, tests to determine which women with DCIS will develop invasive disease remain in development [3], and trials to determine the clinical effectiveness and patient-derived endpoints of active surveillance for DCIS are in progress [4–6]. Thus, the standard of care for most women is to undergo lumpectomy, in part to confirm a DCIS diagnosis, with consideration of adjuvant radiation and hormone therapy, or mastectomy, which may entail short- and long-term treatment-related complications [7, 8].

Management of DCIS is challenging for women and their clinicians. Physicians surveyed in England and the United States indicated that explaining DCIS and justifying treatment to women were difficult [9, 10]. Other studies found variation in the language clinicians used to describe DCIS, with many referring to it as cancer, and variation in treatment patterns [11, 12]. Women with DCIS worldwide have reported suboptimal communication, poor health care experiences, and adverse health outcomes [13–17]. In these studies, most women felt they were given unclear and conflicting information about whether they had cancer; were unaware of treatment options and implications; had inaccurate perceptions of the risk of invasive cancer, metastasis, recurrence, and survival; and experienced similar anxiety and depression as women with invasive breast cancer. Despite the challenges reported by patients and physicians, our scoping review of 51 studies published from 1997 to 2016 identified only two studies that developed interventions to support discussions about DCIS [18].

There is an urgent, widespread need to improve patient–clinician communication about DCIS. Patient-centered care (PCC) offers an approach for doing so. PCC is ideally suited for circumstances when there is limited evidence to support decision-making, when treatment outcomes are difficult to predict or may be adverse, or as is the case for DCIS, when two or more treatment options are suitable [19]. PCC addresses patient values and preferences through information sharing, empathy, empowerment, and health promotion [20–24]. McCormack et al. reviewed literature, observed medical encounters, interviewed patients, and engaged a 13-member expert panel to generate a PCC framework specific to cancer patients of 31 sub-domains within six interdependent domains: fostering patient–clinician relationships, exchanging information, addressing patient emotions, managing uncertainty, making decisions, and enabling patient self-management [25]. PCC is a crucial element of high quality care because it has improved patient (knowledge, relationship with providers, service experience, satisfaction, treatment adherence, quality of life; and reduced anxiety, missed work, readmission rates, and mortality) and health system (appropriate health care utilization, cost-effective service delivery) outcomes [26–29].

No prior research has established guidance on PCC for DCIS. Lo et al. and Robinson et al. employed qualitative methods to explore the information needs of women diagnosed with DCIS; however, those studies did not capture the multidimensional nature of PCC or offer insight on the various strategies to support PCC for DCIS [30, 31]. The purpose of this research was to generate national consensus recommendations on strategies required to achieve PCC for DCIS. Broad adoption of those recommendations could lead to improved experiences and outcomes for women with DCIS and their clinicians.

## Methods

### Approach

The Delphi technique, a widely used approach for establishing expert consensus, was used to generate recommendations for strategies that support PCC for DCIS [32–34]. This approach was chosen because we identified little evidence on strategies to achieve PCC for DCIS [18], necessitating a consensus approach. Potential recommendations were derived from our prior research including a review of published literature [18], and interviews with women with DCIS and clinicians who manage DCIS (to be published elsewhere), then rated in an online questionnaire by an expert panel through two rounds. Ratings are anonymous so that panelists are not unduly influenced by others. Conduct and reporting of this research complied with recommendations for the conduct of online surveys [35], and the Conducting and Reporting of Delphi Studies (CREDES) criteria to enhance rigor [36]. A 9-member research team including health services researchers (ARG, RU) and breast cancer surgeons (FCW, NJLH, GG, LH, PM, MLQ, RW) provided input at all stages, further enhancing rigor. The University Health Network Research Ethics Board reviewed and approved this study.

### Expert panel sampling and recruitment

Delphi panels typically include 8 to 12 members [32–34]; however, research shows an increase in Delphi reliability with increasing panel size [37]. We aimed to establish a 30-member clinician panel to achieve multidisciplinary and national representation, more heavily weighted with surgeons since the standard of treatment is surgery [7, 8]. We asked research team members based in different Canadian provinces to nominate surgeons, oncologists (medical, radiation), radiologists, nurses, and patient navigators specializing in breast cancer to achieve national representation. We did not include general practitioners representing primary care because diagnosis and treatment are most often communicated to women with DCIS by specialists. Nominated clinicians were contacted by email on November 29, 2017 with a brief description of the purpose, process, timing and expected commitment, and were asked to confirm their participation. We also invited women to participate since they could provide first-hand input on PCC for DCIS. Women aged 18 years and older treated for DCIS within the past 2 years from 5 provinces who had participated in prior focus groups were sent an email inviting them to complete the survey. We directly contacted women at 2 of 5 sites; at the remaining 3 sites, due to local research ethics board requirements, a site coordinator communicated with women.

### Survey development

Recommendations to be rated by panelists were derived from a prior scoping review of research published from 1997 to 2016 on DCIS communication experiences, needs, and interventions among DCIS patients or clinicians [18]; and qualitative interviews with 46 clinicians and focus groups involving 35 women with DCIS from across Canada (to be published). From results of the scoping review, interviews, and focus groups, ARG and two research assistants independently extracted facilitators and barriers, and suggestions to improve patient–clinician discussions about DCIS. Those were worded as recommendations, and organized in a table according to the McCormack et al. six-domain framework of PCC: fostering clinician–patient relationships, exchanging information, addressing patient emotions, managing uncertainty, making decisions, and enabling patient self-management [25]. This PCC framework was chosen because it was specific to cancer, included the perspectives of women and clinicians, and had been rigorously developed. The table also displayed the source of each recommendation as one or more of scoping review, clinician interviews, or patient focus groups. The recommendation source document was reviewed by the other 8 members of the research team who offered suggestions for refining the wording of recommendations.

### Data collection and analysis

Recommendations were formatted as a Round One survey administered online using Google Forms. The survey prompted respondents to rate each recommendation on a 7-point Likert scale where 1 was strongly disagree and 7 was strongly agree. The survey was comprised of 46 recommendations on 6 web pages corresponding to McCormack et al. PCC categories [25]. Free text options were included for comments on the wording or content of recommendations, and to suggest additional recommendations not already included in the survey. The survey was reviewed by the research team who offered suggestions to refine the wording and clarify of survey instructions, and to identify errors in spelling or survey functionality. An email with a link to the same survey and the recommendation source document was sent to clinician panelists on March 7, 2018, and women with DCIS panelists between April 5, 2018 and May 2, 2018. The survey of women with DCIS was delayed pending completion of focus groups at all five sites. We sent a reminder email at 2 and 4 weeks.

We calculated Likert scale response frequencies for each recommendation, and summarized comments and newly suggested recommendations. Standard Delphi protocol suggests that two rounds of rating with agreement by two-thirds of panelists will prevent respondent fatigue and drop-out [32–34]. We conducted two rounds of rating; however, to yield unequivocal recommendations, more stringent consensus criteria were applied. Strong consensus for inclusion was defined as 80% or more of panelists agreed or strongly agreed by choosing 6 or 7, or 85% or more chose 5 or 6 or 7; strong consensus for exclusion was defined as 80% or more chose 1 or 2 or 3 or 4; with remaining recommendations categorized as unclear consensus.

The Round One summary report of anonymized results, including Likert rating and comments about the recommendation or its wording, was circulated to panelists by email with a link to the Round Two survey formatted similarly to the Round One survey for rating of recommendations that had not yet achieved consensus for inclusion or exclusion. The email was sent to clinician panelists on April 5, 2018 and to women with DCIS panelists on June 11, 2018, followed by a reminder at 2 and 4 weeks. We analyzed and summarized responses in a manner similar to Round One. Ultimately, items were categorized as recommendations if retained by both women and clinicians, additional considerations if retained by women only, and exclusions if they did not achieve consensus among either women or clinicians.

## Results

### Respondents

Of 49 clinician nominees, 31 accepted the invitation; a total of 32 women were invited to complete the survey. Table 1 summarizes panel composition by province, including 27 women who completed the Round One survey, 11 surgeons, 2 medical oncologists, 4 radiation oncologists, 6 radiologists, and 7 nurses or patient navigators. A total of 29 (96.7%) clinicians and 27 (84.4%) women contacted responded to the Round One survey, and 28 (93.3%) clinicians and 22 of 27 (81.5%) women responded to the Round Two survey.

**Table 1:** Expert panel composition

Panelist category	Province					Subtotal (*n*)
	British Columbia	Alberta	Saskatchewan	Ontario	Nova Scotia	
Women	8	3	3	8	5	27^a^
Clinicians	6	7	4	5	8	30
Surgeons	2	3	1	3	2	11
Medical oncologists	–	–	1	–	1	2
Radiation oncologists	2	2	–	–	–	4
Radiologists	1	–	1	2	2	6
Nurses or navigators	1	2	1	–	3	7
Subtotal (*n*)	14	10	7	13	13	57

^a^Completed round one survey

### Initial recommendations

Supplementary File 1 presents all recommendations to support PCC for DCIS that emerged from prior research (*n* = 46) organized by PCC domains: fostering patient–physician relationship (*n* = 6), exchanging information (*n* = 11), responding to patient emotions (*n* = 3), managing uncertainty (*n* = 4), making decisions (*n* = 13), and enabling patient self-management (*n* = 9). The majority of recommendations were derived from clinician interviews (40, 87.0%) followed by patient focus groups (33, 71.7%) and the scoping review (10, 21.7%). A total of 8 (17.4%) recommendations were common to all three sources; 19 (41.3%) were common to both patients and clinicians. More recommendations were derived from clinicians for exchanging information (clinicians 11, patients 7), managing uncertainty (clinicians 4, patients 2), and making decisions (clinicians 13, patients 9). More recommendations were derived from patients for responding to emotions (patients 3, clinicians 1) and enabling self-management (patients 8, clinicians 7).

### Delphi results

**Fig. 1:**
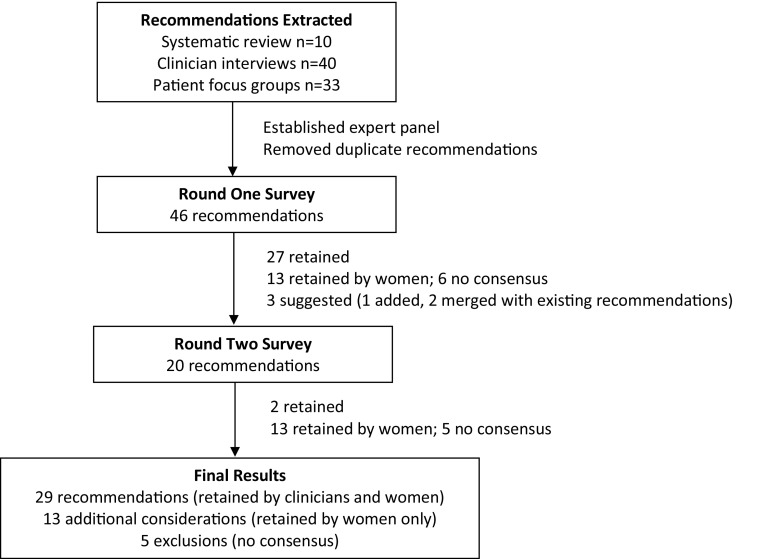
Delphi process and results

Supplementary File 2 presents the rating results of Round One and Round Two. Figure 1 shows the number of recommendations included and excluded in each Round. In Round One, 27 of 46 recommendations were retained by all panelists. The Round Two survey included 20 recommendations: 13 retained by women only and 6 that did not achieve consensus in Round One, plus 1 newly suggested recommendation. Table 2 shows the final results. Twenty-nine recommendations were retained by both women and clinicians in the PCC domains of fostering patient–physician relationship (5), exchanging information (5), responding to emotions (1), managing uncertainty (4), making decisions (9), and enabling patient self-management (5). An additional 13 recommendations were retained by women only: fostering patient–physician relationship (1), exchanging information (3), responding to emotions (2), making decisions (3), and enabling patient self-management (4). A total of 5 recommendations did not achieve consensus among women or clinicians and were excluded.

**Table 2:** DCIS PCC recommendations

PCC domain	Recommendations
Fostering patient–physician relationship Establishing a friendly, courteous, and comfortable relationship	Clinicians should discuss diagnosis and treatment with patients in a non-rushed fashion to foster trust Male clinicians should ensure that a female (i.e., clinician, staff, companion) is present during consultations for patients who express discomfort with male-only interaction Clinicians should encourage questions during and after the first meeting Clinicians should offer undecided patients the option of a repeat discussion of diagnosis and treatment Clinicians should inform patients of next steps and the timing of next steps prior to leaving the first meeting Patients should be offered the opportunity to provide voluntary feedback about the quality of care they receive during and upon conclusion of their treatment (women only)
Exchanging information Words or language used to discuss DCIS	A consensus guideline should be developed to establish the language that clinicians should use when describing DCIS Clinicians should discuss diagnosis and treatment with patients using layman terms if the patient has no clinical background Clinicians should provide patients with pamphlets (or other paper or electronic resource) to take home to further facilitate understanding of DCIS Clinicians should involve a translator in consultations with patients who may have language barriers to understanding DCIS if such resources are available Clinicians should check if patients understand what DCIS is, and the meaning of terms used to describe DCIS, and identify and address inaccurate perceptions Clinicians should use diagrams during consultations to facilitate patient understanding of DCIS (women only) A communication aid should be developed and used to help patients and clinicians discuss DCIS (women only) Family doctors referring patients to specialists should ensure patients are aware of their diagnosis before seeing the specialist (women only)
Responding to patient emotions Response to or managing emotional reaction	Clinicians should acknowledge that a diagnosis of DCIS can be stressful and evoke an emotional response Clinicians should encourage patients access emotional support including counseling and support groups even if patient do not seem outwardly emotional (women only) Clinicians should have a patient navigator or nurse available during or at the end of an appointment to answer questions, help patients process information, and provide information for support groups (women only)
Managing uncertainty Describing likelihood of DCIS turning into invasive cancer or likely prognosis	Conversations about DCIS should include information and/or statistics about the risk of recurrence, metastasis, progression to invasive disease, and prognosis The risk of recurrence or progression with and without additional therapy should be quantified and presented in absolute terms over a 10- or 20-year time frame Clinicians should mention the possibility of invasive disease that biopsy may not detect when there is a reasonable possibility of sampling error Surgeons and oncologists should work closely together so that each conveys to the same patient consistent information about treatment options and risks
Making decisions Involvement in discussing and/or choosing treatment	Clinicians should recommend a treatment option but explain why the option is best suited to patient and tumor characteristics Clinicians should ask questions about lifestyle and views about risks/outcomes to gain a better understanding about patient preferences Clinicians and patients should work together to discuss the merits of treatment options and jointly make a decision about the best option but ultimately it is the patient’s decision to make Clinicians should give patients a week to make a treatment decision Surgeons should refer patients before surgery for consultation with a radiation oncologist if considering lumpectomy, and offer referral to a plastic surgeon if considering mastectomy or lumpectomy Clinicians should explain that, even though patients may want mastectomy or prophylactic mastectomy, it may not be necessary Conversations about treatment options should include information about possible side effects that may occur after treatment such as worsened body image, anxiety, or depression A guideline of DCIS treatment options should be developed to facilitate patient–clinician discussions Educational resources should be made available for DCIS patients considering reconstruction after mastectomy Clinicians should explain that, even though DCIS is not cancer, treatment is necessary to achieve a bigger margin and prevent progression to invasive cancer if applicable to patient (women only) Clinicians may employ a decision aid when discussing treatment options with patients (women only) Regional breast centers should be developed that provide patients with access to various treatment options and supportive care resources so that treatment decisions are not based on avoiding travel time and associated costs (women only)
Enabling patient self-management Setting expectations for follow-up; preparing for self-managing health and well-being	Patients should be aware of their follow-up plan before leaving the care of their surgeon Clinicians should provide patients with pamphlets on routine aftercare including exercise to aid in recovery Websites/external resources should offered to patients who seek more information on DCIS Clinicians should encourage patients to seek emotional support if needed at any point post-DCIS diagnosis and treatment A web site should be developed that lists credible online resources and organizations from which patients can acquire information or support DCIS-specific resources (i.e., pamphlets, support groups) should be developed and offered to patients (women only) Patients should be offered the opportunity to be linked with a patient navigator to provide information and education about DCIS (women only) A card with contact information for patient navigators (and other supportive resources), if available, should be provided to patients to address further questions (women only) Survivorship programs that accept or are specific to DCIS should be developed and offered (women only)

### Future implications

Table 3 lists actionable implications inferred from the recommendations to support PCC for DCIS including the development of a consensus guideline regarding labels and language to use when discussing DCIS, a clinical guideline on DCIS treatment options, a communication aid to support patient–clinician discussions about DCIS, a decision aid to support patient engagement in treatment decision-making, a follow-up plan “prescription” template, and information material that patients can take home that enable self-management and the seeking of additional information or support.

**Table 3:** Future implications for policy and practice

PCC domain	Strategy reflecting women and clinician recommendations	Strategy reflecting women only additional considerations
Fostering patient–physician relationship	–	–
Exchanging information	Develop a consensus guideline on the labels and language that clinicians should use when discussing DCIS Develop information material that patients can take home to further facilitate understanding of DCIS	Develop a communication aid including diagrams to help patients and clinicians discuss DCIS The communication aid could include the following items retained as recommendations Lay language that clinicians should use when the patient has no clinical background Prompts for clinicians to check patient understanding and address inaccurate perceptions Acknowledge stress that can invoke an emotional response
Responding to patient emotions	–	The communication aid could encourage women to seek emotional support through counseling or support group The communication aid could include the following items retained as recommendations Acknowledge stress that can invoke an emotional response
Managing uncertainty	The communication aid could include the following recommendations Information and/or statistics about the risk of recurrence, metastasis, progression to invasive disease, and prognosis Risk should be quantified and presented in absolute terms over a 10- or 20-year time frame Possibility of invasive disease that biopsy may not detect in the likelihood of sampling error	–
Making decisions	Develop a guideline of DCIS treatment options Clinicians and patients should work together to discuss the merits of treatment options and jointly make a decision about the best option, but ultimately it is the patient’s decision to make (*Note* use of decision aid retained by women only)	Develop a decision aid If applicable to the patient, the decision aid should explain that even though DCIS is not cancer, treatment can achieve a bigger margin and prevent progression to invasive cancer The decision aid could include the following items retained as recommendations Mastectomy or prophylactic mastectomy may not be necessary Possible side effects that may occur after treatment including worsened body image, anxiety, or depression Prompts for clinicians to ask about lifestyle, and views about risks and outcomes to better understand patient preferences Prompts for clinicians to explain why a particular treatment option is best suited to the patient and tumor characteristics
Enabling patient self-management	Develop information material on routine aftercare that could also include Contact details for credible web sites or organizations for women who seek more information on DCIS Encouragement to access counseling or support groups if needed at any point through survivorship Develop a follow-up plan “prescription” template Develop a web site that lists credible online resources and organizations from which women can acquire information or support	Develop DCIS-specific information material, patient navigation, supportive care, support groups, or survivorship programs

## Discussion

This research generated national consensus recommendations on strategies to achieve PCC for DCIS, including 29 recommended by both women and clinicians, and 13 additional considerations endorsed by women only. Many recommendations, organized in the PCC domains of fostering a patient–physician relationship, exchanging information, responding to emotions, managing uncertainty, making decisions, and enabling patient self-management, refer to processes during the clinical consultation. Other recommendations refer to informational material or tools that could be used during or after consultation.

Despite the benefits associated with PCC, and insight on the elements of PCC and how to achieve it, many patients do not experience PCC. A national survey in the United States in 2011 showed that, among 2718 responding adults aged 40 or greater with ten common medical conditions, there was considerable variation in whether patients experienced PCC [38]. Suboptimal PCC was reported by half of 1794 American cancer survivors responding in 2013 to a national survey [39]. A survey of 30,849 patients affiliated with 56 primary care sites in one Veterans Health Administration Region before and after medical home (model of coordinated, team-based primary care) implementation between 2010 and 2012 found no improvement in PCC [40]. Therefore, insight is needed on how to achieve PCC. This may be particularly important for women due to gendered disparities in access to and quality of care. In 2016, a Commonwealth Fund national survey revealed that women were less likely than men to have medical needs addressed, access to a specialist, or report good patient–provider communication [41, 42]. A meta-review (28 reviews 2011–2017) identified patient (i.e., tailoring care to values and preferences, providing self-management information, offering emotional support) and professional (i.e., education and training) interventions to achieve PCC [43]. However, that review pertained to patients with various medical conditions. Our study was unique in that it generated insight on how to achieve PCC specifically for DCIS. These recommendations for PCC for DCIS supplement and are complementary to clinical quality indicators for DCIS diagnosis, radiology, treatment, and pathology developed by modified Delphi technique [44]. Together, the clinical quality indicators and PCC recommendations can be used by organizations that deliver or oversee health care to plan services, or evaluate and improve services.

A key next step recommended by panelists was a separate consensus process to establish language that clinicians should use when describing DCIS, although consensus was not achieved on whether to refer to DCIS as something other than cancer. Research has found that significantly more women chose surgery when DCIS was referred to as non-invasive cancer compared with breast lesion or abnormal cells, women are increasingly choosing mastectomy and bilateral mastectomy rather than lumpectomy even though these treatments do not improve breast cancer-specific survival, and clinicians may be driven to over-diagnose and over-treat DCIS due to fear of litigation or missing disease, and feeling compelled to do something rather than nothing [45, 46]. Hence, changing the label for DCIS may be a strategy that avoids over-treatment or, until ongoing trials demonstrate the clinical effectiveness of active surveillance for DCIS [4–6], at the very least reduces confusion and anxiety among women diagnosed with DCIS, and concern about explaining DCIS among clinicians. Precedence for changing labels has been established for bladder, cervical, and thyroid cancers [46].

Another important next step recommended by panelists was to develop resources that support communication, reduce confusion and anxiety, and improve well-being following treatment. These included information for patients on DCIS pathobiology, natural history, treatment options, outcomes, and aftercare; a communication tool to support patient–clinician discussions; a patient decision aid; a “prescription” template detailing the clinical follow-up plan; and a web site listing credible online DCIS resources. We found two DCIS decision aids: one developed in Australia in 2010 for patients although it is not known if the content reflects all aspects of PCC considered important by women [47], and one developed in the United States specifically for use by clinicians as a risk calculator [48]. However, while decision aids support patient engagement in their own care [49], numerous patient, clinician, and system-level barriers limit the implementation and impact of decision aids [50]. Therefore, ongoing research is needed to develop these recommended resources and test their impact on PCC and other outcomes.

This study featured both strengths and limitations. Recommendations reflected the views of multidisciplinary clinicians and women treated for DCIS representing different geographic regions from across Canada. Recommendations were evidence- and consensus-based because they were drawn from a scoping review [18], and primary research involving interviews with clinicians and focus groups with women (to be published elsewhere). We optimized the Delphi process by using a large panel who were identified by nomination [37], and by using only two rounds to prevent respondent fatigue [32–34], and thus achieved relatively high response rates. We complied with research and reporting standards for online surveys [35], and Delphi studies [36]. A 9-member research team reviewed recommendations at all stages, further enhancing rigor. A few issues may limit the interpretation and use of these findings. We did not discuss findings among panelists as is done for the modified Delphi process [32–34], which may have altered the number or nature of final recommendations. Participating women were volunteers, and their views on PCC may differ from other patients. Panelists may reflect the views of Canadian women with DCIS and clinicians and/or the characteristics of Canada’s publicly funded health care system, so recommendations may not apply elsewhere. However, globally women have reported dissatisfaction and confusion with PCC for DCIS [13–17], and clinicians also reported that discussing DCIS with women is challenging [11, 12], so these recommendations to support PCC for DCIS are likely broadly relevant.

In conclusion, a national consensus process involving women with DCIS and multidisciplinary clinicians who specialize in breast cancer generated recommendations for improving PCC for DCIS including the need for communication tools, and a separate consensus process to establish labels and language that clearly and accurately describe DCIS.

## Electronic supplementary material

Below is the link to the electronic supplementary material.

Supplementary material 1 (DOCX 15 KB)

Supplementary File 1. Recommendations extracted from primary sources (DOCX 21 KB)

Supplementary File 2. Delphi results (DOCX 20 KB)
