# Supplementary material for: National consensus recommendations on patient-centered care for ductal carcinoma in situ
Source: Breast Cancer Res Treat. 2019 Jan 9;174(3):561–70. doi: 10.1007/s10549-019-05132-z (PMC6438938; doi:10.1007/s10549-019-05132-z)
Supplement: Supplementary file 2 — Supplementary File 1. Recommendations extracted from primary sources (DOCX 21 KB) [file 10549_2019_5132_MOESM2_ESM.docx]

National consensus recommendations on patient-centered care for ductal carcinoma in situ

Breast Cancer Research & Treatment

Anna R Gagliardi, Frances C Wright, Nicole J Look Hong, Gary Groot, Lucy Helyer, Pamela Meiers, May Lynn Quan, Robin Urquhart, Rebecca Warburton (Corresponding: Anna R Gagliardi, University Health Network, Toronto, Canada, anna.gagliardi@uhnresearch.ca)

**Supplementary File 1. Recommendations extracted from primary sources**

**Fostering Patient-Physician Relationship**

*Establishing a friendly, courteous and comfortable relationship*

| Indicator | Source | | | Common to all 3 sources | Common to clinicians and patients |
| --- | --- | --- | --- | --- | --- |
|  | Systematic review | Clinician interviews | Patient focus groups |  |  |
| Clinicians should discuss diagnosis and treatment with patients in a non-rushed fashion to foster trust |  |  | X |  |  |
| Male clinicians should ensure that a female clinician is present during consultations for patients that express discomfort with male-only interaction |  | X |  |  |  |
| Patients should be asked to give feedback on the quality of care they receive |  | X |  |  |  |
| Clinicians should encourage questions during and after the first meeting |  | X | X |  | X |
| Clinicians should be accessible by phone or email to answer questions and concerns after the first meeting |  | X | X |  | X |
| Clinicians should inform patients of next steps and the timing of next steps prior to leaving the first meeting |  |  | X |  |  |
| Subtotal (n) | 0 | 4 | 4 | 0 | 2 |

**Exchanging Information**

*Words or language used to explain DCIS*

| Indicator | Source | | | Common to all 3 sources | Common to clinicians and patients |
| --- | --- | --- | --- | --- | --- |
|  | Systematic review | Clinician interviews | Patient focus groups |  |  |
| Clinicians should emphasize that DCIS is not cancer and non-invasive |  | X | X |  | X |
| Clinicians should use the term abnormal cells rather than cancer, carcinoma, pre-invasive cancer or stage 0 cancer when referring to DCIS | X | X | X | X |  |
| The terminology “DCIS” should be changed to exclude the word “carcinoma” |  | X | X |  | X |
| A consensus guideline should be developed to establish the language that clinicians should use when describing DCIS |  | X |  |  |  |
| Clinicians should discuss diagnosis and treatment with patients using lay language |  | X | X |  | X |
| Clinicians should use diagrams during consultations to facilitate patient understanding of DCIS |  | X | X |  | X |
| A communication aid should be developed to help patients and clinicians discuss DCIS | X | X |  |  |  |
| Clinicians should provide patients with pamphlets (or other paper or electronic resource) to take home to further facilitate understanding of DCIS |  | X |  |  |  |
| Clinicians should involve a translator in consultations with patients who may have language barriers to understanding DCIS |  | X |  |  |  |
| Clinicians should check if patients understand what DCIS is, and the meaning of terms used to describe DCIS , and identify and address inaccurate perceptions | X | X | X | X |  |
| Family doctors referring patients to specialists should ensure patients are aware of their diagnosis before seeing the specialist |  | X | X |  | X |
| Subtotal (n) | 3 | 11 | 7 | 2 | 5 |

**Responding to Patient Emotions**

*Response to or management of emotional reaction*

| Indicator | Source | | | Common to all 3 sources | Common to clinicians and patients |
| --- | --- | --- | --- | --- | --- |
|  | Systematic review | Clinician interviews | Patient focus groups |  |  |
| Clinicians should acknowledge the emotional experience common to most patients diagnosed with DCIS |  |  | X |  |  |
| Clinicians should offer empathy and emotional support even if patients do not seem outwardly emotional |  |  | X |  |  |
| Clinicians should have a patient navigator or nurse available during or at the end of an appointment to answer questions and help patients process information |  | X | X |  | X |
| Subtotal | 0 | 1 | 3 | 0 | 1 |

**Managing Uncertainty**

*Describing likelihood of DCIS turning into invasive cancer or likely prognosis*

| Indicator | Source | | | Common to all 3 sources | Common to clinicians and patients |
| --- | --- | --- | --- | --- | --- |
|  | Systematic review | Clinician interviews | Patient focus groups |  |  |
| Conversations about DCIS should include information and/or statistics about the risk of: recurrence, metastasis, progression to invasive disease, and dying from DCIS | X | X | X | X |  |
| Clinicians should emphasize the low risk of progression to invasive disease and of dying from breast disease |  | X | X |  | X |
| Clinicians should mention the possibility of invasive disease that biopsy may not detect |  | X |  |  |  |
| If applicable to a given patient, surgeons, radiation oncologists, and medical oncologists should work closely together so that each conveys to patients the same information about treatment options and risks |  | X |  |  |  |
| Subtotal (n) | 1 | 4 | 2 | 1 | 1 |

**Making Decisions**

*Involvement in discussing and/or choosing treatment*

| Indicator | Source | | | Common to all 3 sources | Common to clinicians and patients |
| --- | --- | --- | --- | --- | --- |
|  | Systematic review | Clinician interviews | Patient focus groups |  |  |
| Clinicians should encourage breast conserving surgery with radiation over mastectomy and specify recurrence rates associated with treatment options | X | X | X | X |  |
| Clinicians should recommend a treatment option but explain why the option is best suited to a woman’s age, tumor grade, demographic characteristics, etc. | X | X | X | X |  |
| Clinicians should ask questions about lifestyle and views about risks/outcomes to gain a better understanding about patient preferences |  | X | X |  | X |
| Clinicians and patients should work together to discuss the merits of treatment options and jointly make a decision about the best option | X | X | X | X |  |
| Clinicians should give patients time to make a treatment decision and ensure that patient is 100% comfortable with the treatment decision |  | X | X |  | X |
| Surgeons should refer patients for consultation with a radiation oncologist if considering lumpectomy and a plastic surgeon if considering mastectomy before finalizing treatment decisions |  | X | X |  | X |
| Radiologists should see patients on two occasions – for diagnosis and prior to surgery – but should defer the discussion of treatment options to surgeons or specialists |  | X |  |  |  |
| Clinicians should explain that, even though DCIS is not cancer, invasive treatment is necessary to achieve a bigger margin and prevent progression to invasive cancer |  | X | X |  | X |
| Clinicians should explain that, even though patients may want more aggressive treatment, it isn’t necessary |  | X | X |  | X |
| Conversations about treatment options should include information about possible side effects that may occur after treatment such as worsened body image, identity, future risk, tension, nervousness, loneliness, anxiousness, and depression | X | X | X | X |  |
| A guideline of DCIS treatment options should be developed to facilitate patient-clinician discussions |  | X |  |  |  |
| Clinicians should employ a decision aid when discussing treatment options with patients |  | X |  |  |  |
| Regional breast centres should be developed that provide patients with access to various treatment options and supportive care resources so that treatment decisions are not based on avoiding travel time and associated costs |  | X |  |  |  |
| Subtotal (n) | 4 | 13 | 9 | 4 | 5 |

**Enabling Patient Self-Management**

*Setting expectations for follow-up; preparing for self-managing health and well-being*

| Indicator | Source | | | Common to all 3 sources | Common to clinicians and patients |
| --- | --- | --- | --- | --- | --- |
|  | Systematic review | Clinician interviews | Patient focus groups |  |  |
| Patients should be aware of their follow-up plan before leaving the care of their surgeon |  |  | X |  |  |
| Clinicians should provide patients with pamphlets on routine after-care |  | X | X |  | X |
| DCIS-specific resources (i.e. supportive care, support groups) should be developed and offered to patients |  | X | X |  | X |
| Websites/external resources should offered to patients who seek more information on DCIS | X | X | X | X |  |
| Patients should be linked with a patient navigator to provide information and education about DCIS |  | X | X |  | X |
| Patients should be given a card with contact information for patient navigators (and other supportive services) to address further questions |  | X | X |  | X |
| Clinicians should acknowledge that patients went through a profound experience and encourage them to seek psychological help at any point throughout survivorship |  |  | X |  |  |
| A web site should be developed that lists credible online resources and organizations from which patients can acquire information or support | X | X |  |  |  |
| Survivorship programs that accept or are specific to DCIS should be developed and offered |  | X | X |  | X |
| Subtotal (n) | 2 | 7 | 8 | 1 | 5 |
