# Supplementary material for: National consensus recommendations on patient-centered care for ductal carcinoma in situ
Source: Breast Cancer Res Treat. 2019 Jan 9;174(3):561–70. doi: 10.1007/s10549-019-05132-z (PMC6438938; doi:10.1007/s10549-019-05132-z)
Supplement: Supplementary file 3 — Supplementary File 2. Delphi results (DOCX 20 KB) [file 10549_2019_5132_MOESM3_ESM.docx]

National consensus recommendations on patient-centered care for ductal carcinoma in situ

Breast Cancer Research & Treatment

Anna R Gagliardi, Frances C Wright, Nicole J Look Hong, Gary Groot, Lucy Helyer, Pamela Meiers, May Lynn Quan, Robin Urquhart, Rebecca Warburton (Corresponding: Anna R Gagliardi, University Health Network, Toronto, Canada, [anna.gagliardi@uhnresearch.ca](mailto:anna.gagliardi@uhnresearch.ca))

**Supplementary File 2. Delphi results**

| Domain | Recommendation | Suggested revision | Round 1 | | Round 2 | | Retained by both |
| --- | --- | --- | --- | --- | --- | --- | --- |
|  |  |  | Clinicians | Women | Clinicians | Women |  |
| Fostering patient-physician relationship  *Establishing a friendly, courteous and comfortable relationship* | Clinicians should discuss diagnosis and treatment with patients in a non-rushed fashion to foster trust | --- | Retain | Retain | --- | --- | Retain |
|  | Male clinicians should ensure that a female health professional is present during consultations for patients that express discomfort with male-only interaction | Male clinicians should ensure that a female (i.e. clinician, staff, companion) is present during consultations for patients who express discomfort with male-only interaction | Retain | Retain | --- | --- | Retain |
|  | Patients should be asked to give feedback on the quality of care they receive upon conclusion of their treatment | Patients should be offered the opportunity to provide voluntary feedback about the quality of care they receive during and upon conclusion of their treatment | No consensus | Retain | No consensus | --- | Retain-patients |
|  | Clinicians should encourage questions during and after the first meeting | --- | Retain | Retain | --- | --- | Retain |
|  | Clinicians should offer undecided patients the option of returning for a second visit to re-discuss diagnosis and treatment | Clinicians should offer undecided patients the option of a repeat discussion of diagnosis and treatment | Retain | Retain | --- | --- | Retain |
|  | Clinicians should inform patients of next steps and the timing of next steps prior to leaving the first meeting | --- | Retain | Retain | --- | --- | Retain |
| Sub-total |  |  | 5 | 6 | 0 | --- | 5 |
| Exchanging information  *Words or language used to explain DCIS* | Clinicians should emphasize that DCIS is not cancer and non-invasive | --- | No consensus | No consensus | No consensus | No consensus | No consensus |
|  | Clinicians should use the term abnormal cells rather than cancer, carcinoma, pre-invasive cancer or stage 0 cancer when referring to DCIS | --- | No consensus | No consensus | No consensus | No consensus | No consensus |
|  | The terminology “DCIS” should be changed to exclude the word “carcinoma” | --- | No consensus | No consensus | Discard | No consensus | No consensus |
|  | A consensus guideline should be developed to establish the language that clinicians should use when describing DCIS | *We will regard this as a recommendation for future research/initiative* | Retain | Retain | --- | --- | Retain |
|  | Clinicians should discuss diagnosis and treatment with patients using lay language | Clinicians should discuss diagnosis and treatment with patients using layman terms if the patient has no clinical background | Retain | Retain | --- | --- | Retain |
|  | Clinicians should use diagrams during consultations to facilitate patient understanding of DCIS | --- | No consensus | Retain | No consensus | --- | Retain-patients |
|  | A communication aid should be developed to help patients and clinicians discuss DCIS | A communication aid should be developed/used to help patients and clinicians discuss DCIS | No consensus | Retain | No consensus | --- | Retain-patients |
|  | Clinicians should provide patients with pamphlets (or other paper or electronic resource) to take home to further facilitate understanding of DCIS | --- | Retain | Retain | --- | --- | Retain |
|  | Clinicians should involve a translator in consultations with patients who may have language barriers to understanding DCIS | Clinicians should involve a translator in consultations with patients who may have language barriers to understanding DCIS if such resources are available | Retain | Retain | --- | --- | Retain |
|  | Clinicians should check if patients understand what DCIS is, and the meaning of terms used to describe DCIS , and identify and address inaccurate perceptions | --- | Retain | Retain | --- | --- | Retain |
|  | Family doctors referring patients to specialists should ensure patients are aware of their diagnosis before seeing the specialist | --- | No consensus | Retain | No consensus | --- | Retain-patients |
| Sub-total |  |  | 5 | 8 | 0 | 0 | 5 |
| Responding to Patient Emotions  *Response to or management of emotional reaction* | Clinicians should acknowledge that most women experience an emotional reaction when diagnosed with DCIS | Clinicians should acknowledge that a diagnosis of DCIS can be stressful and evoke an emotional response | Retain | Retain | --- | --- | Retain |
|  | Clinicians should encourage patients to seek psychological support even if patients do not seem outwardly emotional | Clinicians should offer patients access to resources or a referral for psychological support even if patients do not seem outwardly emotional.  Clinicians should encourage patients access emotional support including counselling and support groups even if patient do not seem outwardly emotional | No consensus | No consensus | No consensus | Retain | Retain-patients |
|  | Clinicians should have a patient navigator or nurse available during or at the end of an appointment to answer questions and help patients process information | Clinicians should have patient navigator or nurse available during or at the end of an appointment, if possible, to answer questions and help patient’s process information.  Clinicians should have a patient navigator or nurse available during or at the end of an appointment to answer questions, help patients process information and provide information for support groups | No consensus | Retain | No consensus | --- | Retain-patients |
| Sub-total |  |  | 1 | 2 | 0 | 1 | 1 |
| Managing Uncertainty  *Describing likelihood of DCIS turning into invasive cancer or likely prognosis* | Conversations about DCIS should include information and/or statistics about the risk of: recurrence, metastasis, progression to invasive disease, and dying from DCIS | Conversations about DCIS should include information and/or statistics about the risk of: recurrence, metastasis, progression to invasive disease, and prognosis | Retain | Retain | --- | --- | Retain |
|  | Clinicians should emphasize the low risk of progression to invasive disease and of dying from breast disease | The risk of recurrence or progression with and without additional therapy should be quantified and presented in absolute terms over a 10- or 20-year time frame  Clinicians should mention the low risk of progression to invasive disease and of dying from breast disease | Retain | No consensus | --- | Retain | Retain |
|  | Clinicians should mention the possibility of invasive disease that biopsy may not detect | Clinicians should mention the possibility of invasive disease that biopsy may not detect when there is a reasonable possibility of sampling error  Clinicians should mention that the disease may be more extensive than what the biopsy shows | Retain | Retain | --- | --- | Retain |
|  | If applicable to a given patient, surgeons, radiation oncologists, and medical oncologists should work closely together so that each conveys to patients the same information about treatment options and risks | Surgeons and oncologists should work closely together so that each conveys to the same patient consistent information about treatment options and risks | Retain | Retain | --- | --- | Retain |
| Sub-total |  |  | 4 | 3 | 0 | 1 | 4 |
| Making Decisions  *Involvement in discussing and/or choosing treatment* | Clinicians should encourage breast conserving surgery with radiation over mastectomy and specify recurrence rates associated with treatment options | Clinicians should encourage breast conserving surgery with/without radiation over mastectomy and specify recurrence rates associated with treatment options | No consensus | No consensus | No consensus | No consensus | No consensus |
|  | Clinicians should recommend a treatment option but explain why the option is best suited to patient and tumour characteristics | --- | Retain | Retain | --- | --- | Retain |
|  | Clinicians should ask questions about lifestyle and views about risks/outcomes to gain a better understanding about patient preferences | --- | Retain | Retain | --- | --- | Retain |
|  | Clinicians and patients should work together to discuss the merits of treatment options and jointly make a decision about the best option | Clinicians and patients should work together to discuss the merits of treatment options and jointly make a decision about the best option but ultimately it is the patient’s decision to make | Retain | Retain | --- | --- | Retain |
|  | Clinicians should give patients time to make a treatment decision | Clinicians should give patients a week to make a treatment decision | Retain | Retain | --- | --- | Retain |
|  | Surgeons should refer patients before or after surgery for consultation with a radiation oncologist if considering lumpectomy, and offer referral to a plastic surgeon if considering mastectomy | Surgeons should refer patients before surgery for consultation with a radiation oncologist if considering lumpectomy, and offer referral to a plastic surgeon if considering mastectomy or lumpectomy | Retain | Retain | --- | --- | Retain |
|  | Radiation oncologists should see patients on two occasions – for diagnosis and prior to surgery – but should defer the discussion of treatment options to surgeons | Radiation oncologists (i.e. clinicians who specialize in radiation therapy) should see patients on two occasions – for diagnosis and prior to surgery – but should defer the discussion of treatment options to surgeons | Discard | No consensus | --- | No consensus | No consensus |
|  | Clinicians should explain that, even though DCIS is not cancer, treatment is necessary to achieve a bigger margin and prevent progression to invasive cancer | Clinicians should explain that, even though DCIS is not cancer, treatment is necessary to achieve a bigger margin and prevent progression to invasive cancer if applicable to patient | No consensus | Retain | No consensus | --- | Retain-patients |
|  | Clinicians should explain that, even though patients may want mastectomy or prophylactic mastectomy, it may not be necessary | --- | Retain | Retain | --- | --- | Retain |
|  | Conversations about treatment options should include information about possible side effects that may occur after treatment such as worsened body image, anxiety or depression | --- | Retain | Retain | --- | --- | Retain |
|  | A guideline of DCIS treatment options should be developed to facilitate patient-clinician discussions | --- | Retain | Retain | --- | --- | Retain |
|  | Clinicians should employ a decision aid when discussing treatment options with patients | Clinicians may employ a decision aid when discussing treatment options with patients | No consensus | Retain | No consensus | --- | Retain-patients |
|  | Regional breast centres should be developed that provide patients with access to various treatment options and supportive care resources so that treatment decisions are not based on avoiding travel time and associated costs | --- | No consensus | Retain | No consensus | --- | Retain-patients |
| Sub-total |  |  | 8 | 11 | 0 | 0 | 8 |
| Enabling Patient Self-Management  *Setting expectations for follow-up; preparing for self-managing health and well-being* | Patients should be aware of their follow-up plan before leaving the care of their surgeon | --- | Retain | Retain | --- | --- | Retain |
|  | Clinicians should provide patients with pamphlets on routine after-care | --- | Retain | Retain | --- | --- | Retain |
|  | DCIS-specific resources (i.e. supportive care, support groups) should be developed and offered to patients | --- | No consensus | Retain | No consensus | --- | Retain-patients |
|  | Websites/external resources should offered to patients who seek more information on DCIS | --- | Retain | Retain | --- | --- | Retain |
|  | Patients should be linked with a patient navigator or nurse to provide information and education about DCIS | Patients should be offered the opportunity to be linked with a patient navigator to provide information and education about DCIS. | No consensus | Retain | No consensus | --- | Retain-patients |
|  | A card with contact information for patient navigators (and other supportive resources) should be provided to patients to address further questions | A card with contact information for patient navigators (and other supportive resources) should be provided to patients to address further questions if available. | No consensus | Retain | No consensus | --- | Retain-patients |
|  | Clinicians should encourage patients to seek psychological support if needed at any point throughout survivorship | Clinicians should encourage patients to seek emotional support if needed at any point post-DCIS | Retain | Retain | --- | --- | Retain |
|  | A web site should be developed that lists credible online resources and organizations from which patients can acquire information or support | --- | Retain | Retain | --- | --- | Retain |
|  | Survivorship programs that accept or are specific to DCIS should be developed and offered | --- | No consensus | Retain | No consensus | --- | Retain-patients |
| Sub-total |  |  | 5 | 9 | 0 | 0 | 5 |
| Indicators suggested in Round One and rated in Round Two | Satisfaction (or decisional regret) with DCIS management decision should be assessed 1 year after diagnosis | Satisfaction (or decisional regret) with DCIS management decision should be assessed at follow up visit or 1 year after diagnosis for study purposes. | --- | --- | No consensus | --- |  |
|  | Educational resources should be made available for DCIS patients considering reconstruction after mastectomy |  | --- | --- | Retain | --- |  |
|  | At the one year follow up clinicians should advise how often future mammograms should be scheduled for preventive measures |  | --- | --- | --- | Retain |  |
|  | Exercise after surgery should be encouraged to aid with recovery |  | --- | --- | --- | Retain |  |
